# *Notes from the Field:* First Reports of Locally Transmitted Seoul Hantavirus Infection — District of Columbia, May 2018–December 2018

**DOI:** 10.15585/mmwr.mm7109a3

**Published:** 2022-03-04

**Authors:** Nivedita Ravi-Caldwell, Preetha Iyengar, John Davies-Cole

**Affiliations:** 1 Division of Epidemiology-Disease Surveillance and Investigation, Center for Policy, Planning and Evaluation, District of Columbia Department of Health.

In May 2018, patient A, a previously healthy man aged 30 years, was evaluated at a District of Columbia (DC) health care facility for a 4-day history of chills, diarrhea, fever (103°F [39.5°C]), headache, sore throat, and vomiting. Despite symptomatic treatment with antipyretics, he subsequently experienced hemoconcentration (hematocrit = 60.4% [normal = 38.8%–50%]), thrombocytopenia (<10,000 platelets/*μ*L [normal = 150,000–450,000]), and acute kidney injury (blood urea nitrogen [BUN] = 95 mg/dL [normal = 9–20] and creatinine = 4.95 mg/dL [normal = 0.66–1.50]) over several days (*1*). Approximately 1 week later, he experienced signs consistent with hemophagocytic lymphohistiocytosis, a potentially fatal systemic inflammatory syndrome, including elevated ferritin, triglycerides, and interleukin-2 levels, and hemophagocytosis cells on bone marrow biopsy (*2*). Patient A was a maintenance worker with frequent rodent sightings at his workplace. Serology results were positive for hantavirus immunoglobulin (Ig) M and IgG. Additional tests sent to CDC returned with hantavirus IgG and IgM titers >1:6,400, confirming recent infection with Seoul hantavirus (SEOV). Virus isolation was unsuccessful (*1*). Comprehensive testing for other infectious etiologies returned negative results. The patient responded to supportive treatment and was eventually discharged.

Five months later, in November 2018, patient B, a man aged 37 years with history of chronic kidney disease, was evaluated in a DC emergency department with a 3-day history of chills, fever (104°F [40.1°C]), headache, myalgia, and productive cough, followed later by diarrhea, nausea, and vomiting. On further evaluation, he had elevated hepatic transaminases (aspartate aminotransferase = 164 units/L [normal = 3–34], alanine aminotransferase = 78 units/L [normal = 15–41]), thrombocytopenia (43,000/*μ*L), and acute kidney injury (BUN = 36 mg/dL and creatinine = 7.6 mg/dL) during his admission. The patient worked as a dishwasher and plumber’s assistant, had no recent history of travel outside the United States, and did not own any pets. He was unaware of exposure to rodents at work, at home, or during his commute. Serology sent to rule out hemorrhagic fever-renal syndrome returned with IgG and IgM titers of >1:6,400, confirming recent infection with SEOV. Comprehensive testing for other infectious etiologies returned negative results. The patient responded to supportive treatment and was eventually discharged.

SEOV is a type of hantavirus previously associated with hemorrhagic fever-renal syndrome.* Patient A is believed to have had the first case of hemophagocytic lymphohistiocytosis related to hantavirus infection reported in the United States and the second worldwide (*1*,*3*). Past studies have documented that Norway rats serve as the reservoir species for SEOV in the United States (*1*,*4*), and previous cases of hantavirus infection have been linked to wild or pet rodents (*4*,*5*). Humans can become infected with SEOV through aerosol exposure to virus shed in rodent feces, saliva, or urine. Rodent overpopulation in DC is well documented by increased complaints via the Citywide Call Center to the Rodent Control Program, and the DC Department of Health has amplified efforts to address this public health threat.^†^ Although extremely rare, the two SEOV cases presented in this report highlight the importance of physicians including hantavirus infection in their differential diagnoses in patients with compatible symptoms and history of animal exposure or travel and underscore the importance of reporting notifiable infectious disease cases to health departments for investigation and response. These cases also serve as a reminder to the public to minimize risk for infection by following recommended hygiene practices.^§^

## Footnotes

* https://www.cdc.gov/hantavirus/hfrs/index.html (Accessed June 21, 2019).

†
https://www.washingtonpost.com/graphics/2018/local/rat-calls/

§
https://www.cdc.gov/hantavirus/outbreaks/seoul-virus/cleaning-up-pet-rodents.html

## References

[1] Shastri B, Kofman A, Hennenfent A, Domestically acquired Seoul virus causing hemophagocytic lymphohistiocytosis—Washington, DC, 2018. Open Forum Infect Dis 2019;6:ofz404. 10.1093/ofid/ofz404 31660366 PMC6790396

[2] Rouphael NG, Talati NJ, Vaughan C, Cunningham K, Moreira R, Gould C. Infections associated with haemophagocytic syndrome. Lancet Infect Dis 2007;7:814–22. 10.1016/S1473-3099(07)70290-6 18045564 PMC7185531

[3] Lee J-J, Chung I-J, Shin D-H, Hemorrhagic fever with renal syndrome presenting with hemophagocytic lymphohistiocytosis. Emerg Infect Dis 2002;8:209–10. 10.3201/eid0802.010299 11897077 PMC2732452

[4] Easterbrook JD, Kaplan JB, Vanasco NB, A survey of zoonotic pathogens carried by Norway rats in Baltimore, Maryland, USA. Epidemiol Infect 2007;135:1192–9. 10.1017/S0950268806007746 17224086 PMC2870671

[5] Knust B, Brown S, de St Maurice A, Multistate Seoul Virus Outbreak Investigation Team. Seoul virus infection and spread in United States home-based ratteries: rat and human testing results from a multistate outbreak investigation. J Infect Dis 2020;222:1311–9. 10.1093/infdis/jiaa307 32484879

